# Does PARP Inhibition Sensitize Chondrosarcoma Cell Lines to Chemotherapy or Radiotherapy? Results From a Three-dimensional Spheroid Cell Model

**DOI:** 10.1097/CORR.0000000000002483

**Published:** 2022-12-13

**Authors:** Ieva Palubeckaitė, Sanne Venneker, Brendy E. W. M. van den Akker, Inge H. Briaire-de Bruijn, Judith V. M. G. Boveé

**Affiliations:** 1 Department of Pathology, Leiden University Medical Center, Leiden, the Netherlands

## Abstract

**Background:**

Chondrosarcomas are well known for their resistance to conventional chemotherapy and radiotherapy treatment regimens, which is particularly detrimental in patients who have unresectable tumors. Recently, inhibition of poly(ADP-ribose) polymerase (PARP) by talazoparib was shown to sensitize chondrosarcoma cell lines to chemotherapy (temozolomide) or radiotherapy, irrespective of isocitrate dehydrogenase (IDH) mutation status. Because two-dimensionally grown cell lines have limitations and may not accurately represent the clinical response to drug treatment, we aimed to use a more representative three-dimensional alginate spheroid chondrosarcoma model. It is important to test therapeutic agents in vitro before testing them in animals or humans; therefore, we aimed to determine the effectiveness of a PARP inhibitor in reducing the viability of chondrosarcoma spheroids. Using a more stringent, complex in vitro model refines future therapeutic options for further investigation in animal models, increasing efficiency, reducing unnecessary animal use, and saving time and cost.

**Questions/purposes:**

(1) Does talazoparib treatment slow or inhibit the growth of chondrosarcoma spheroids, and does an increased treatment duration change the drug’s effect? (2) Does talazoparib work in synergy with temozolomide treatment to reduce the viability of chondrosarcoma spheroids? (3) Does talazoparib work in synergy with radiotherapy treatment to reduce the viability of chondrosarcoma spheroids?

**Methods:**

Three representative conventional chondrosarcoma cell lines (CH2879 [IDH wildtype], JJ012 [IDH1 mutant], and SW1353 [IDH2 mutant]) were cultured as alginate spheroids and treated with talazoparib (0.001 to 10 µM), temozolomide (0.01 to 100 µM), or combinations of these drugs for 3, 7, and 14 days, representing different stages of spheroid growth. The cell lines were selected to represent a variety of IDH mutation statuses and were previously validated in spheroid culturing. Temozolomide was chosen because of its previous success when combined with PARP inhibitors, dissimilar to other commonly used chemotherapies. The effect on spheroid viability was assessed using three cell viability assays. Additionally, spheroid count, morphology, proliferation, and apoptosis were assessed. The effect of talazoparib (5 to 10 nM) combined with ƴ-radiation applied using a ^137^C source (0 to 6 Gy) was assessed as surviving fractions by counting the number of spheroids (three). The therapeutic synergy of low-concentration talazoparib (5 to 10 nM) with temozolomide or radiotherapy was determined by calculating Excess over Bliss scores.

**Results:**

Talazoparib treatment reduced the spheroid viability of all three cell lines after 14 days (IC_50_ ± SD of CH2879: 0.1 ± 0.03 µM, fold change: 220; JJ012: 12 ± 1.4 µM, fold change: 4.8; and SW1353: 1.0 ± 0.2 µM, fold change: 154), compared with 3-day treatments of mature spheroids. After 14 days of treatment, the Excess over Bliss scores for 100 µM temozolomide and talazoparib indicated synergistic efficacy (Excess over Bliss scores: CH2879 59% [lower 95% CI 52%], JJ012 18% [lower 95% CI 8%], and SW1353 55% [lower 95% CI 25%]) of this combination treatment. A stable synergistic effect of talazoparib and radiotherapy was present only in JJ012 spheroids at a 4Gƴ radiation dose (Excess over Bliss score: 22% [lower 95% CI 6%]).

**Conclusion:**

In our study, long-term PARP inhibition was more effective than short-term treatment, and only one of the three chondrosarcoma spheroid lines was sensitive to combined PARP inhibition and radiotherapy. These findings suggest subsequent animal studies should focus on long-term PARP inhibition, and temozolomide combined with talazoparib has a higher chance of success than combination with radiotherapy.

**Clinical Relevance:**

Combination treatment of talazoparib and temozolomide was effective in reducing the viability of chondrosarcoma spheroids and spheroid growth, regardless of IDH mutation status, providing rationale to replicate this treatment combination in an animal chondrosarcoma model.

## Introduction

Chondrosarcomas are a heterogeneous group of bone sarcomas that are characterized by the formation of cartilage by tumor cells, with variable clinical behavior leading to an approximate 5-year survival rate of 74% to 99% for patients with Grade II chondrosarcoma and 31% to 77% for those with Grade III chondrosarcoma [10]. Conventional chemotherapy and radiotherapy have very limited efficacy in patients with advanced chondrosarcoma, and surgical resection is the only curative treatment option [2, 37]. Therefore, there is an urgent need to select therapeutic strategies with a higher likelihood of success in treating patients with advanced chondrosarcoma. One of the alternative treatments currently considered for patients is inhibition of poly(ADP-ribose) polymerase (PARP), because its sensitivity is linked to isocitrate dehydrogenase (IDH) mutations, which are common in patients with chondrosarcoma [1]. A recent clinical trial included five patients who had chondrosarcoma with tumors harboring IDH1/2 mutations; preliminary activity of PARP inhibition was observed [17].

Typically, in vitro therapeutic preclinical testing is performed using cell lines in traditional confluent two-dimensional (2D) cell cultures. However, it is unlikely that candidates for oncology therapy will be approved, and better preclinical in vitro selection is required to discard less-promising therapy candidates early on to reduce time, cost, and the number of animal models required for validation [8, 22]. Specifically, advancement of in vitro models of bone malignancies, such as chondrosarcoma, would increase the success of subsequent clinical trials by filtering out unsuccessful treatment options. Alternative in vitro cancer models consist of three-dimensional (3D) cell culture constructs such as multicellular tumor spheroids or hydrogels, which are intended to recapitulate the tumor microenvironment to a greater extent. More-complex in vitro models are particularly relevant when studying sarcoma because of the presence of an extracellular matrix and high physiochemical heterogeneity in the microenvironment, which is not present in conventional 2D cultures [7, 16, 21]. Previously, our group developed and fully characterized a simple-to-use alginate hydrogel spheroid model of chondrosarcoma [31]. Alginate clonal spheroids are created by mixing single-cell suspensions with alginic acid solution and exposing them to an excess of calcium to gelate the solution into a solid hydrogel bead. Over time, these single cells grow into clonal spheroids and produce a glycosaminoglycan and collagen II–rich matrix that is characteristic of chondrosarcoma. The treatment response was altered in chondrosarcoma alginate spheroid models compared with conventional 2D cultures, including changes in synergy when therapeutic combinations were used. Moreover, alginate spheroids could be used for multiple-dose treatments, which proved useful for monitoring treatment resistance and studying therapies requiring longer observation, such as radiotherapy. Generally, the response observed in the alginate spheroid model better represented current in vivo and clinical outcomes [31].

Based on the effect observed in IDH-mutant glioma and leukemia, in which the IDH mutation caused vulnerability to PARP inhibition, talazoparib, a PARP inhibitor currently approved to treat patients with germline BRCA-mutated (gBRCA) HER2-negative metastatic breast cancer, was tested in chondrosarcoma using conventionally 2D-cultured endogenous IDH-mutant and IDH-wildtype cell lines [27, 39]. Sensitivity to talazoparib was, however, variable, and no difference was observed between IDH-mutant and IDH-wildtype cell lines [39]. Instead, talazoparib treatment sensitized chondrosarcoma cell lines to temozolomide or radiation, irrespective of the IDH mutation status. Because conventionally grown cell lines are limited with respect to representativity, we wished to use our matrix-rich 3D chondrosarcoma alginate spheroid model to determine which novel treatment strategies should be further explored in animal models. The conventional culture model was able to give some information, such as a lack of synergy with other chemotherapies such as cisplatin and doxorubicin; however, the most effective approach could be elucidated further by using 3D cell culture models before animal studies.

Using a 3D chondrosarcoma alginate spheroid model, we asked: (1) Does talazoparib treatment slow or inhibit the growth of chondrosarcoma spheroids, and does an increased treatment duration change the drug’s effect? (2) Does talazoparib work in synergy with temozolomide treatment to reduce the viability of chondrosarcoma spheroids? (3) Does talazoparib work in synergy with radiotherapy treatment to reduce the viability of chondrosarcoma spheroids?

## Materials and Methods

### Experimental Overview

**Fig. 1:**
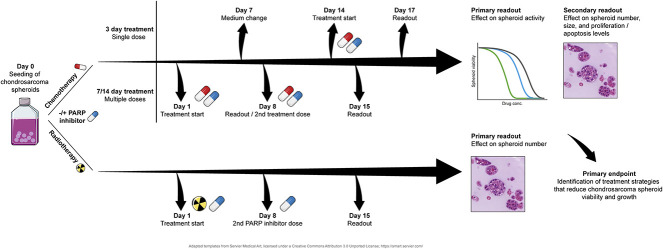
Alginate spheroids were produced by suspending single chondrosarcoma cells within an alginate scaffold and culturing them in either multiplexed culture flasks or 96-well plates when treatment was applied. Visible spheroid growth can be observed after 7 days. The 7-day and 14-day treatments were administered on Day 1 and Day 8, and samples were harvested on Days 8 and 15, respectively. The 3-day treatment was administered to samples after 14 days of growth, and samples were harvested on Day 17. The primary assay performed to assess talazoparib and temozolomide treatment was the Presto Blue cell activity assay. The primary assay to assess radiation treatment and combination with talazoparib was a 3D colony-formation assay. Synergy was assessed based on these datasets. Cell morphology, spheroid number, proliferation, and apoptosis were additionally assessed.

To more effectively predict chondrosarcoma’s sensitivity to talazoparib and talazoparib combined with either temozolomide or radiotherapy, we used therapeutic response assays to assess an alginate spheroid model, partially bridging the gap between in vitro and in vivo models (Fig. 1). The study was split into two culture regimens: 3-day treatment of mature (14-day-old) spheroids and 7-day and 14-day treatment of spheroids in cultures. The response to talazoparib and temozolomide was measured primarily by a cell viability assay during each treatment. These results were used to quantify therapeutic sensitivity and calculate the synergy of the drug combination. The response to talazoparib and radiotherapy was measured primarily using a 3D colony-formation assay. Therapeutic sensitivity was primarily determined from spheroid count and is presented as surviving fractions.

### Cell Culture

CH2879 (Grade III, IDH1: G105G [wt]) [19], JJ012 (Grade II, IDH1: R132G mut) [23, 34], and SW1353 (Grade II, IDH2: R172S mut) (ATCC) conventional chondrosarcoma cell lines were cultured in RPMI 1640 medium (Life Technologies Limited) containing 10% fetal bovine serum and 50 U/mL of penicillin and 50 μg/mL of streptomycin (Life Technologies Limited) [15, 32]. These lines were previously selected for alginate spheroid models because they provide a varied selection of tumor grades and IDH mutation statuses. Because of the more laborious nature of 3D cell culture experiments, a larger panel of cell lines was not used. The cultures were maintained in a humidified atmosphere containing 5% CO_2_ at 37°C. A short tandem repeat analysis was performed before and after the experiments to confirm the identity of the cell lines, using the Cell ID Gene Print 10 system (Promega Benelux BV). The negativity of mycoplasma was confirmed regularly.

Cell lines were transferred to an alginate bead culture as described elsewhere [31]. The cells were cultured in Dulbecco modified eagle medium, nutrient mixture F-12, for a maximum of 17 days.

### Assessment of Response to Antineoplastic Agents

Temozolomide (S1237, Selleckchem) and talazoparib (BMN-673, PARP1/2 inhibitor) (S7048, Selleckchem) were selected to treat chondrosarcoma spheroids and dissolved in dimethyl sulfoxide according to the manufacturer’s instructions. Clinically relevant ranges of temozolomide (0.01 to 100 µM) or talazoparib (0.001 to 10 µM) were administered alone or in combination. When therapies were combined, the concentration of talazoparib was kept stable (5 nM for CH2879 and 10 nM for the other cell lines) and a range of temozolomide concentrations was used (0.01 to 100 µM). Temozolomide is currently approved for newly diagnosed glioblastoma multiforme and refractory anaplastic astrocytoma and can reach plasma concentrations of approximately 72 µM after multiple daily doses of up to 200 mg/m^2^ per day [11, 30]. Talazoparib is currently approved for gBRCA HER2-negative metastatic breast cancer, reaching plasma concentrations of approximately 55 nM after multiple daily doses of up to 1 mg per day [14]. The combination of temozolomide and talazoparib was successful in reducing the viability of 2D-cultured chondrosarcoma cells [39]. These results were similar to those of multiple studies that successfully combined temozolomide and PARP inhibitors, providing a rationale for further testing in an alginate spheroid model [20, 28, 40].

To assess sensitivity to talazoparib and temozolomide, dose-response curves were generated for the three cell lines in 3D at 3, 7, and 14 days of treatment, as described in previous work [31]. Briefly, the Day 3 treatments were performed using mature (14-day-old) spheroids, whereas treatments for 7 or 14 days were administered throughout spheroid growth starting on Day 1. Spheroid growth during this period was investigated, and cultures were determined to be in the growth phase until the latest endpoint of 17 days, after which growth slowed as cells reached their maximum size [31]. The alginate cultures were seeded as single cells at Day 0, growing to ≤ 50 µm in diameter at 7 days and ≤ 200 µm at 14 days (Supplementary Fig. 1; http://links.lww.com/CORR/A982). Sensitivity to talazoparib and temozolomide was determined by quantifying cell activity using the Presto Blue assay (Invitrogen, Life Technologies) according to the manufacturer’s instructions. After incubation with the Presto Blue reagent, culture plates were measured with an Infinite M Plex microplate reader (Tecan). The readout was used to determine the drug concentration that inhibited cell viability by 50% (IC_50_) and to determine synergy of the combination therapy. The fold differences in IC_50_ values for the 3-, 7-, and 14-day treatments were compared. The data analysis was performed using Prism software version 7.02 after normalization to untreated control samples. The experiments were performed three times. The Bliss independence model (C = A + B – A × B) was used to quantify synergy, in which C represents the combined effect and A and B represent single-agent effects [9]. Synergy is defined as a marked increase in effectiveness when therapies are used in combination, compared with the summed effect of monotherapies. The resulting values indicate synergy at > 0, additive effect at 0, and antagonistic effect at < 0. Heatmap figures were created with MORPHEUS for improved visualization (Broad Institute). The resulting synergy values were only defined as reliable if the lower 95% confidence interval (CI) value was also above the additive effect threshold of 0.

### Assessment of Radiation Therapy Response

To observe sensitivity to radiotherapy, colony-formation assays are typically used because clonogenic capacity is reduced after irradiation. Because of the clonal formation of alginate spheroids, these can be treated as colonies for assessing radiation. Alginate spheroids were used for radiation treatment experiments, as described [31]. Increasing doses (0 to 6 Gy) of γ-radiation using a ^137^C source (YXLON, Comet Technologies) were applied to culture plates containing cell-seeded alginate beads, along with varying concentrations of talazoparib. Talazoparib was administered 2 hours before radiotherapy to correspond to current clinical trial regimens [25]. Spheroids were cultured for 14 days, with a refresh of the treatment medium at Day 8, then fixed in formalin and embedded in paraffin. The spheroid number within an area spanning multiple alginate beads was quantified for the three experiments. Changes in the surviving fraction after 2-Gy (SF2) treatment are considered to indicate the in vivo radiotherapy response [18]. Significant changes in SF2 were investigated by comparing fold changes. Using surviving fraction results, the Bliss independence model was used to quantify the synergy of talazoparib treatment combined with radiotherapy (as described under “Assessment of Response to Antineoplastic Agents”). The resulting synergy values were tested for significant discrepancy by checking the 95% CI values were above the additive effect value of 0.

### Histologic Staining

Hematoxylin and eosin staining was used to facilitate qualitative morphologic observation after treatment with talazoparib and temozolomide, as well as quantitative counting of spheroids after radiation with or without talazoparib. Treated spheroids were fixed using 10% neutral buffered formalin, dehydrated, and embedded in paraffin. Sections were stained with hematoxylin and eosin using the Leica ST5020-CV5030 Stainer Integrated Workstation (Leica Biosystems), and an expert bone tumor pathologist (JVMGB) performed a morphologic assessment. The total colony number and size were counted in a representative area spanning multiple alginate beads using QuPath open-source software [6]. This was performed in triplicate for the radiotherapy dataset because this was the primary outcome. For the talazoparib and temozolomide samples, spheroid count was used as additional supporting data; therefore, only one randomly selected batch of samples was assessed.

### Immunohistochemistry

To approximate the effect of talazoparib and temozolomide on proliferation (Ki67) and apoptosis (cleaved caspase-3) levels in alginate spheroids, we stained for specific markers in a subset of samples. For Ki67 (anti-Ki67 D2H10 rabbit monoclonal [1:1600]) (9027S, Cell Signaling) and activated caspase-3 (anti-activated caspase-3 rabbit polyclonal [1:800)]) (9661S, Cell Signaling), immunohistochemical staining was performed, as described [31]. Scanning was performed using a Pannoramic 250 Flash III scanner (3D Histech) using 40X magnification. Positive and negative controls were used for each antibody for quality control.

The scanned IHC samples were scored using QuPath open-source software [6]. All scorings were performed in a representative area spanning multiple alginate beads. Ki-67 was scored using the software’s positive cell detection tool to determine the percentage of positive nuclei in a representative area. Activated caspase-3 staining was scored by manually counting the total percentage of positive cells in a representative area. The dose-response curve experiments were performed three times; however, to calculate the percentages of Ki67 and activated caspase-3, we randomly selected one batch and considered the scores of this one experiment as representative.

## Results

### Talazoparib Sensitivity and Treatment Duration Effects

**Fig. 2:**
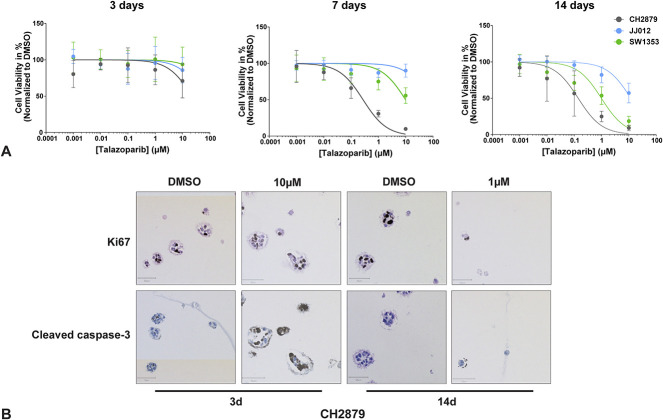
Chondrosarcoma spheroids are more sensitive to PARP inhibition after a longer treatment duration. (**A**) Dose-response curves of talazoparib after 3 days of treatment of mature spheroids or 7 or 14 days of treatment of growing spheroids for three chondrosarcoma cell lines indicate the effect of talazoparib is increased with treatment time. The datapoints represent the mean of three experiments performed in triplicate ± standard deviation. (**B**) The number and size of spheroids decreased after 14 days of talazoparib treatment compared with 3 days of treatment or dimethyl sulfoxide–treated controls. A small fraction of proliferative cells persisted after 14 days of treatment. CH2879 spheroids were used to represent trends.

The alginate spheroid viability was reduced in all three cell lines after talazoparib treatment, particularly after 14 days of treatment. A dose-dependent decrease in the viability of chondrosarcoma spheroids with an increase in treatment time was observed for all three chondrosarcoma cell lines (Fig. 2A). Fourteen-day treatment led to a 220-fold reduction in IC_50_ compared with 3-day treatment for the CH2879 spheroids (IC_50_ mean ± SD = 22 ± 5.5 µM versus 0.1 ± 0.03 µM) (Table 1), a 4.8-fold reduction in IC_50_ for JJ012 spheroids (IC_50_ mean ± SD = 58 ± 22 µM versus 12 ± 1.4 µM) (Table 2), and a 154-fold reduction in IC_50_ for SW1353 spheroids (IC_50_ mean ± SD = 154 ± 151 µM versus 1.0 ± 0.2 µM) (Table 3). To confirm the cell viability findings, a subset of samples (one) was used for hematoxylin and eosin and IHC staining to determine the number of spheroids and approximate levels of proliferation and apoptosis in each condition. The cell viability results were confirmed; spheroid numbers were reduced after 14 days of treatment with 1 µM of talazoparib compared with the untreated control in all three cell lines, in contrast to 3-day treatment with 10 µM of talazoparib, in which a lack of cell viability response was confirmed by the lack of reduction in spheroid number (Supplementary Fig. 2; http://links.lww.com/CORR/A983). Observation of proliferation and apoptosis levels in this subset of samples also confirmed the viability results, because no marked change was observed in proliferation after 3 days, contrary to 14-day treatment, although increased apoptosis was observed in two cell lines (Supplementary Fig. 3; http://links.lww.com/CORR/A986). This can be clearly visualized in the CH2879 spheroids (Fig. 2B).

**Table 1.** Inhibitory concentration (IC_50_) parameters ± standard deviation for talazoparib, temozolomide monotherapies, and combination treatments of CH2879 spheroids

Treatment	3 days (mature)	7 days	14 days	Fold change (3 days vs 14 days)
Talazoparib, µM	22 ± 5.5	0.3 ± 0.1	0.1 ± 0.03	220
Temozolomide, µM	n/e	n/e	1125 ± 583	n/e
Combination, μM (temozolomide + 5 nM talzoparib)	926 ± 471	81 ± 12	59 ± 7.1	16
Fold change (temozolomide vs combination)	n/e	n/e	19	

Data are provided as mean ± SD. n/e = not evaluable; IC_50_ = half maximal inhibitory concentration. Fold changes are for temozolomide monotherapy versus combination as well as between 3-day (mature spheroids) and 14-day treatments.

**Table 2.** Inhibitory concentration (IC_50_) parameters ± standard deviation for talazoparib, temozolomide monotherapies and combination treatments of JJ012 spheroids.

Treatment	3 days (mature)	7 days	14 days	Fold change (3 days vs 14 days)
Talazoparib, µM	58 ± 22	76 ± 24	12 ± 1.4	4.8
Temozolomide, µM	750 ± 183	812 ± 281	307 ± 52	2.4
Combination, µM (temozolomide + 10 nM talazoparib)	8878 ± 29,226	447 ± 88	136 ± 18	65
Fold change (temozolomide vs combination)	0.1	1.8	2.3	

Data are provided as mean ± SD. n/e = not evaluable; IC_50_ = half maximal inhibitory concentration. Fold changes for temozolomide monotherapy versus combination as well as between 3-day (mature spheroids) and 14-day treatments are given.

**Table 3.** Inhibitory concentration (IC_50_) parameters ± standard deviation for talazoparib, temozolomide monotherapies and combination treatments of SW1353 spheroids.

Treatment	3 days (mature)	7 days	14 days	Fold change (3 days vs 14 days)
Talazoparib, µM	154 ± 151	10 ± 2.2	1.0 ± 0.2	154
Temozolomide, µM	n/e	896 ± 413	431 ± 227	n/e
Combination, µM (temozolomide + 10 nM talazoparib)	761 ± 581	344 ± 64	19 ± 6.1	40
Fold change (temozolomide vs combination)	n/e	2.6	23	

Data are provided as mean ± SD. n/e = not evaluable; IC_50_ = half maximal inhibitory concentration. Fold changes for temozolomide monotherapy versus combination as well as between 3-day (mature spheroids) and 14-day treatments are given.

### Talazoparib’s Synergy With Temozolomide

**Fig. 3:**
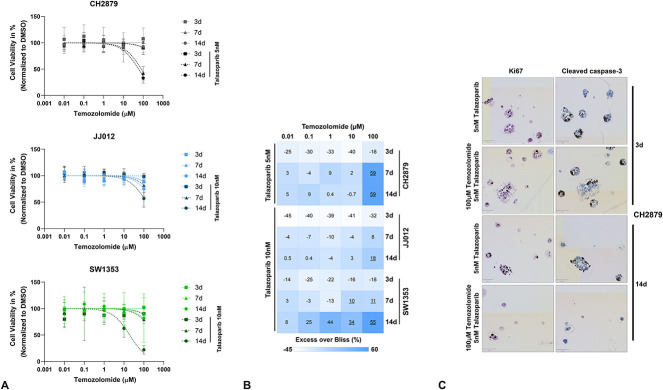
A combination of PARP inhibition and temozolomide is synergistic in chondrosarcoma spheroids. (**A**) Dose-response curves for single temozolomide treatment or combination treatment indicate that talazoparib sensitized all cell lines to temozolomide. The datapoints represent the mean of three experiments in triplicate ± standard deviation. (**B**) This heatmap represents the calculated Excess over Bliss scores for temozolomide and talazoparib combinations, in which a value below zero represents antagonism (white), a value of zero represents additivity (light blue), and a value above zero represents synergy (blue). The underlined Excess over Bliss scores are qualified for statistical inference (lower 95% CI above 0). Talazoparib combined with 100 µM of temozolomide was synergistic in all tested cell lines when treated for 14 days. (**C**) IHC staining of Ki67 (proliferation) and cleaved caspase-3 (apoptosis) was performed. No difference in proliferation and apoptosis levels was observed after combination treatment of CH2879 spheroids.

The combination of talazoparib and temozolomide was more effective on the alginate spheroids than both treatments together, indicating a synergistic effect. Low-dose (5 or 10 nM) talazoparib combined with temozolomide led to reduced cell viability, as opposed to the use of temozolomide alone in all three cell lines (Fig. 3A). Temozolomide treatment alone had no effect on CH2879 spheroids for all treatment durations, whereas combination with 5 nM of talazoparib led to a 19-fold reduction in IC_50_ after 14 days of treatment (IC_50_ mean ± SD = 1125 ± 583 µM to 59 ± 7.1 µM) (Table 1). JJ012 spheroids showed a slight response to temozolomide monotherapy after longer treatment (more than 7 days); however, combination with talazoparib (10 nM) was still beneficial because it led to a 2.3-fold reduction in IC_50_ after 14 days (IC_50_ mean ± SD = 307 ± 52 µM to 136 ± 18 µM) (Table 2). Combination treatment of the SW1353 spheroids led to a 23-fold reduction in IC_50_ compared with monotherapy after 14 days of treatment (IC_50_ mean ± SD = 431 ± 227 µM to 19 ± 6.1 µM) (Table 3). The synergistic effect was the greatest for 100 µM of temozolomide combined with talazoparib for 14 days (Excess over Bliss scores: CH2879 59% [lower 95% CI 52%], JJ012 18% [lower 95% CI 8%], and SW1353 55% [lower 95% CI 25%]), although the combination was already synergistic after 7 days of treatment for CH2879 and SW1353 spheroids (Fig. 3B). Other Excess over Bliss scores indicating a synergistic treatment effect (> 0), were not confirmed, because the lower 95% CI for these was below the threshold of 0 (Supplementary Fig. 4; http://links.lww.com/CORR/A987). As was done for talazoparib monotherapy samples, to confirm the cell viability findings, a subset of samples (one) was used for hematoxylin and eosin and IHC staining to determine the number of spheroids and approximate levels of proliferation and apoptosis in each condition. Similar to talazoparib monotherapy, the cell viability results were confirmed in a subset of samples by an observed decrease in number of spheroids after 14 days of treatment (Supplementary Fig. 5; http://links.lww.com/CORR/A988). No large changes in apoptotic or proliferative fractions of CH2979 were observed after treatment (Fig. 3C). Proliferation and apoptosis were quantified in a subset of samples, indicating this more clearly for all three spheroid lines (Supplementary Fig. 6; http://links.lww.com/CORR/A989). A lack of apoptosis despite clear reduction in cell viability would indicate these cells are dying via other mechanisms, such as autophagy. In this subset of samples, JJ012 spheroids exhibited higher proliferation levels after combination treatment compared with talazoparib monotherapy. This could indicate outgrowth of a resistant subpopulation during treatment, which should be investigated in future experiments.

### Talazoparib’s Synergy With Radiotherapy

**Fig. 4:**
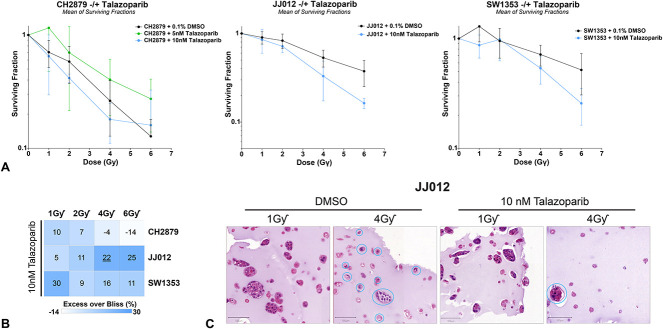
PARP inhibition sensitized chondrosarcoma spheroids to radiation. (**A**) This figure shows surviving fractions of three chondrosarcoma cell lines after treatment with single doses of γ-radiation and 5 or 10 nM talazoparib. Talazoparib sensitized JJ012 spheroids toward radiotherapy. (**B**) This heatmap represents the calculated Excess over Bliss scores for radiation and talazoparib combinations, in which a value below zero represents antagonism (white), a value of zero represents additivity (light blue), and a value above zero represents synergy (blue). Underlined Excess over Bliss scores are qualified for statistical inference (lower 95% CI above 0). Combination treatment induced synergy in the JJ012 cell line at high radiation doses and SW1353 at a 1 Gƴ dose. (**C**) Hematoxylin and eosin–stained images of radiotherapy and talazoparib combination–treated JJ012 spheroids indicate decreased spheroid number and size when combining with higher-dose radiation. The blue circles highlight remaining spheroids with cells containing intact nuclei to differentiate these from remaining dead spheroid debris in the samples with a higher radiation dose.

Combined talazoparib with one-dose radiation therapy led to a visible reduction in the surviving fraction of JJ012 spheroids, while the other two cell lines showed varied results (Fig. 4A). However, none of the spheroid lines had a fold-change reduction in surviving fraction at the clinically relevant 2-Gγ dose (SF2 mean ± SD CH2879 0.4 ± 0.04, fold change: 1.5; JJ012: 0.7 ± 0.1, fold change: 1.1; SW1353: 1.0 ± 0.4, fold change: 1), even with the addition of 10 nM of talazoparib (Table 4). The combination of 10 nM of talazoparib and a 4 Gƴ radiotherapy dose led to a reliable, synergistic effect on JJ012 spheroids (Excess over Bliss score 4 Gƴ 22% [lower 95% CI 6%]), indicating there was therapeutic synergy of talazoparib with radiotherapy (Fig. 4). Other Excess over Bliss scores indicating a synergistic treatment effect (> 0) were not confirmed because the lower 95% CI for these was below the threshold of 0 (Supplementary Fig. 7; http://links.lww.com/CORR/A990*).* The loss of spheroid number and size was visible at the 4-Gƴ dose combination, supporting these results (Fig. 4C).

**Table 4.** Surviving fractions at 2 Gγ (SF2) parameters for radiotherapy with and without talazoparib treatment.

**Treatment**	**CH2879**	**JJ012**	**SW1353**
Radiotherapy + 0.1% DMSO	0.6 ± 0.2	0.8 ± 0.2	1.0 ± 0.3
Radiotherapy + 5 nM talazoparib	0.7 ± 0.5	n/e	n/e
Radiotherapy + 10 nM talazoparib	0.4 ± 0.04	0.7 ± 0.1	1.0 ± 0.4
Fold change (-/+10nM talazoparib)	1.5	1.1	1

Data are provided as mean ± SD. n/e = not evaluable. Fold changes for radiation only versus combination with 10nM talazoparib are given.

## Discussion

Chondrosarcomas are a heterogenous group of malignant cartilaginous bone tumors that are highly resistant to conventional chemotherapy and radiotherapy. Alternative treatment options are sought, including vulnerabilities caused by IDH mutations, which are the most common mutations in chondrosarcoma. Mutations in the IDH1 and IDH2 genes were shown to cause vulnerability to PARP inhibition in other cancer types; however, talazoparib showed variable sensitivity in a panel of conventionally cultured chondrosarcoma cell lines [27, 36, 39]. Importantly, there was no difference in the sensitivity to PARP inhibition between IDH-mutant and IDH-wildtype chondrosarcoma cell lines [39]. Instead, PARP inhibition with talazoparib sensitized chondrosarcoma cell lines to temozolomide or radiation, irrespective of their IDH mutation status. However, because conventional 2D cell culture results often do not correlate well with the findings of animal studies, we opted to first test the treatment regimens using a more-representative 3D chondrosarcoma model to determine whether these novel treatment strategies should be explored further in mouse models of orthotopic chondrosarcoma. Use of a chondrosarcoma spheroid model such as this one is beneficial because of the presence of a cartilaginous matrix as well as more-representative physiochemical gradients and cell attachments that alter the effectiveness of therapy.

We confirmed that single-dose talazoparib monotherapy would most likely be ineffective, and multiple doses over longer time periods will be required to observe a response. We further identified the combination of talazoparib and temozolomide as effective in all three spheroid lines, whereas combination with radiotherapy only worked in one chondrosarcoma spheroid line. Overall, the effective concentration of each therapeutic agent in spheroids was higher than effective concentrations previously found in 2D cultured chondrosarcoma cell lines, providing a higher dose range for further experiments. These findings help refine options for further in vivo testing, improving efficiency and reducing the amount of animals and time required.

### Limitations

Although we showed that 3D chondrosarcoma alginate spheroids are more representative than 2D cell line cultures [31], these models have limitations. Advanced 3D in vitro models are not as simple to use as standard 2D cultures, making large-scale drug screening more difficult. Additionally, the alginate spheroid model still does not fully recapitulate the tumor microenvironment because it lacks key features, such as the presence of vascularization and an immune cell population*.* Although spheroids were shown to produce a cartilaginous matrix, their relatively small size may provide easier drug access than solid tumors in vivo.

The current study used talazoparib for PARP inhibition because this inhibitor has the broadest FDA and European Medicines Agency approval, although olaparib monotherapy could also be considered [13]. Both inhibitors have similar efficacy in patients with gBRCA-mutated HER2-negative metastatic breast cancer; however, they differ in their adverse-effect risk profile [26]. Thus, future studies in chondrosarcoma might also include a PARP inhibitor with a different adverse-effect profile.

The growth rates of the three cell lines differed in alginate spheroid culture, and methods commonly used for correcting 2D cell culture growth are not applicable to 3D. Because CH2879 spheroids had the highest growth rate in our previous study [31], this may partly explain the sensitivity observed in the current study.

### Talazoparib Sensitivity and Treatment Duration Effects

Using this greater-reliability 3D model of alginate spheroid chondrosarcoma, we determined chondrosarcoma spheroid treatment with talazoparib was not effective after 3 days, as observed with conventionally cultured cell lines [39]. However, longer treatment times, particularly 14 days, were effective in inducing loss of cell viability and reducing spheroid numbers. The longer treatment period required for chondrosarcoma spheroids is more in line with the clinical setting, where talazoparib is given continuously until disease progression or unacceptable toxicity occurs [3]. These longer treatment periods cannot be evaluated by conventional 2D culture. In the current study, although spheroids were more therapy-resistant than conventional cultures, the dose required for a treatment response was lower, with longer time periods. Thus, although the monotherapy dose was still higher than in clinically observed plasma concentrations (approximately 26 to 55 nM [14, 41]), the observed sensitivity for PARP inhibition in 2D and 3D chondrosarcoma cultures would support further exploration of PARP inhibition for patients with chondrosarcoma. Sensitivity was unrelated to IDH mutation status, confirming previous findings. Of interest, a recently published Phase II clinical study of PARP inhibition using olaparib as monotherapy reported a clinical benefit in three of five patients with IDH-mutant chondrosarcoma (one patient with a partial response and two with stable disease persisting for more than 7 months) [17]. Only participants with IDH-mutant chondrosarcoma were selected for this trial. Before undertaking further clinical trials, in vivo chondrosarcoma xenograft studies could examine the effects of multiple doses with talazoparib and olaparib in tumors with a mixed selection of IDH mutation statuses. This may reveal whether patients with IDH-wildtype chondrosarcomas could also benefit from PARP inhibitor monotherapy.

### Talazoparib’s Synergy With Temozolomide

To improve the efficacy of PARP inhibition treatment, we further explored its combination with the DNA alkylating and methylating agent temozolomide. Combination treatment was highly synergistic and led to decreased cell viability and spheroid number in all cell lines, compared with talazoparib and temozolomide monotherapies, confirming previous results in 2D [39]. Using a combination treatment allowed for lower talazoparib concentrations that were below the range of clinically observed effective plasma values (approximately 26 to 55 nM) when combined with clinically relevant temozolomide plasma values (approximately 72 µM) [4, 14, 41]. If these results are replicated in chondrosarcoma xenograft studies and are eventually used in humans, reducing the concentration of PARP inhibitors may help avoid side effects including common hematologic toxicities that often lead to dose modification, interruption, and discontinuation [24]. Our results would therefore support further exploration of talazoparib combined with temozolomide in orthotopic chondrosarcoma mouse models. Thus far, clinical trials and animal studies have investigated combinations with low-dose temozolomide. However, our results demonstrated synergy for the opposite regimen (high-dose temozolomide with low-dose talazoparib) [33, 35]. Altered treatment concentrations, in line with these results, may induce a stronger response. Combining the two agents has demonstrated potency against several cancer types; however, animal studies and a recent Phase I/II clinical trial (NCT02116777) have indicated that a dose reduction may be required to make the combination tolerable [35]. Toxicity could be overcome through better formulation strategies, such as a nanoparticle formulation of talazoparib, which was effective in reducing toxicity in xenografts with Ewing sarcoma [5]. In the future, discovery of next-generation selective PARP inhibitors, such as AZD5305, which is currently in clinical development, will further improve the pharmacologic profile and reduce adverse effects [29]. These options could be considered in the design of future animal model studies.

### Talazoparib’s Synergy With Radiotherapy

PARP inhibition was found to increase the sensitivity of conventionally cultured chondrosarcoma cell lines to radiotherapy [12, 39]; therefore, we further investigated potential synergy in this spheroid model. The JJ012 cell line spheroids showed an increased response to radiotherapy when combined with talazoparib treatment; however, dissimilar to previous 2D model studies, the results were not conclusive for the other two spheroid lines. Sensitivity can be explained by a nonclassic DNA damage repair deficiency in JJ012, which was not observed in the other two cell lines, such as a low capacity to sense DNA damage or defects downstream in the homologous recombination pathway, as indicated in a previous study [39]. Our result is contradictory to published data on the CH2879 cell line in which radiotherapy sensitization was observed with PARP inhibition; however, this was largely attributed to a delay in cell proliferation [12]. Cell proliferation is typically lower in 3D in vitro models and in vivo models than in 2D culture. Because of delayed cell proliferation, any subsequent treatment effect may not be as pronounced in 3D cell cultures and animal models, more accurately predicting clinical outcome. Our results indicate this combination treatment may only be effective in a small subset of DNA damage repair-deficient tumors. However, this conclusion is currently only based on a study of three spheroid lines and should be confirmed in larger panels. This outcome was not as clearly defined in previous 2D culture studies, and future studies are needed to confirm treatment efficacy in this subset of chondrosarcomas.

### Conclusion

Using a more-complex chondrosarcoma culture model, we confirmed the sensitivity of chondrosarcoma to talazoparib monotherapy, irrespective of the IDH mutation status, although multiple dosing and longer treatment regimens were required. Moreover, combination with temozolomide was synergistic and enabled a lower treatment dose over a longer treatment duration. Combination with irradiation was synergistic in one of three cell lines, indicating that deficiency in DNA damage repair may be linked to sensitivity. Our study provides the rationale for a further exploration of talazoparib and temozolomide for chondrosarcoma in an orthotopic mouse model, such as the one developed by our group [38], and those studies should assess a combination of low-concentration talazoparib and temozolomide, as well as a combination of temozolomide with recently developed lower-toxicity PARP inhibitor treatment options. A further study of the link to deficiency in DNA damage repair is required before talazoparib combined with radiotherapy is assessed in vivo. If similar findings are observed in xenograft models, these therapies may be worth considering in clinical trials.
